# Barley Nepenthesin-Like Aspartic Protease *HvNEP-1* Degrades *Fusarium* Phytase, Impairs Toxin Production, and Suppresses the Fungal Growth

**DOI:** 10.3389/fpls.2021.702557

**Published:** 2021-07-29

**Authors:** Zelalem Eshetu Bekalu, Giuseppe Dionisio, Claus Krogh Madsen, Thomas Etzerodt, Inge S. Fomsgaard, Henrik Brinch-Pedersen

**Affiliations:** Department of Agroecology, Aarhus University, Slagelse, Denmark

**Keywords:** *Fusarium*, *Hordeum vulgare* L., nepenthesin, rHvNEP-1, fungal phytases, fungal growth, mycotoxin, trichothecene

## Abstract

Nepenthesins are categorized under the subfamily of the nepenthesin-like plant aspartic proteases (PAPs) that form a distinct group of atypical PAPs. This study describes the effect of nepenthesin 1 (*HvNEP-1*) protease from barley (*Hordeum vulgare* L.) on fungal histidine acid phosphatase (HAP) phytase activity. Signal peptide lacking HvNEP-1 was expressed in *Pichia pastoris* and biochemically characterized. Recombinant HvNEP-1 (rHvNEP-1) strongly inhibited the activity of *Aspergillus* and *Fusarium* phytases, which are enzymes that release inorganic phosphorous from phytic acid. Moreover, rHvNEP-1 suppressed *in vitro* fungal growth and strongly reduced the production of mycotoxin, 15-acetyldeoxynivalenol (15-ADON), from *Fusarium graminearum*. The quantitative PCR analysis of trichothecene biosynthesis genes (*TRI*) confirmed that rHvNEP-1 strongly repressed the expression of *TRI4*, *TRI5*, *TRI6*, and *TRI12* in *F*. *graminearum*. The co-incubation of rHvNEP-1 with recombinant *F. graminearum* (rFgPHY1) and *Fusarium culmorum* (FcPHY1) phytases induced substantial degradation of both *Fusarium* phytases, indicating that HvNEP-1-mediated proteolysis of the fungal phytases contributes to the HvNEP-1-based suppression of *Fusarium*.

## Introduction

Nepenthesins are the first group of proteases reported from the nepenthesin-like plant aspartic proteases (PAPs). They represent only the extracellular protease of plant origin. As described for the nepenthesin-like PAPs, nepenthesins are characterized by diverse N-terminal sequence and nepenthesin-type PAP insertion (NAP-I) sequence (Simoes and Faro, 2004; Soares et al., 2019; Bekalu et al., 2020a). They were initially described from the pitcher fluid of the carnivorous plant, *Nepenthes* (Athauda et al., 2004). Later, they have been purified from various carnivorous plant species and characterized *in vitro* (Bekalu et al., 2020a). Their biological function is mainly linked to the degradation of insect proteins as a nitrogen source. Several genes coding for protein homologs to nepenthesins have been identified in *Arabidopsis thaliana* and *Oryza sativa* (Takahashi et al., 2008; Chen et al., 2009). They display a high diversity and widespread tissue expression, suggesting their participation in various physiological processes in plants. In addition to their extended biological roles, nepenthesins have recently been implicated for various industrial applications, for example, as a tool for Digestion in Hydrogen/Deuterium Exchange Mass Spectrometry (Yang et al., 2015), and treatment for celiac disease (Rey et al., 2016).

Phytases are phosphatases essential for initializing the sequential release of orthophosphates from phytic acid, i.e., the main seed storage form of phosphorous (P) in small grain cereals (Boyd et al., 1972; Lott, 1984; Vohra and Satyanarayana, 2003). Plants, animals, and microorganisms are the sources of numerous phytases (Gen Lei et al., 2007). However, while fungal phytases are histidine acid phosphatase (HAP), the main plant phytases belong to purple acid phosphatase phytases (Holme et al., 2017). Fungal pathogens such as *Aspergillus* and *Fusarium* need phytases to access the phytic acid–bound P in the seeds (Marlida et al., 2010; Bhavsar et al., 2012; Gontia-Mishra et al., 2014). Phytases from fungus and bacteria are often used as feed supplements to monogastric animals that lack sufficient endogenous phytase activity (Konietzny and Greiner, 2004; Singh and Satyanarayana, 2015). However, the study reported that bacteria use phytases as a pathogenesis factor to colonize host tissues (Chatterjee et al., 2003). Similarly, pathogenic fungi may also use phytases during the infection of host tissues.

Fungal species from the *Fusarium* species complex produce phosphatases with significant phytase activity (Marlida et al., 2010; Gontia-Mishra et al., 2014; Ferreira et al., 2020). Some phytase-producing *Fusarium* species are important pathogens of crop plants. For instance, *F. graminearum* and *Fusarium culmorum* have been widely known as the main causative agents of Fusarium head blight (FHB), the disease that causes considerable losses of yield and quality in cereals, mainly in temperate regions (Nganje et al., 2004; Stenglein, 2009). FHB is often linked with the accumulation of mycotoxins in the grain that substantially reduce grain quality (Chen et al., 2019). The most abundant mycotoxins include zearalenone (ZEA) and Type B trichothecenes, such as nivalenol (NIV), deoxynivalenol (DON), and its 3-acetyldeoxynivalenol (3-ADON) or 15-ADON. Fifteen *TRI* genes encode for the enzymes involved in the biosynthesis of trichothecene in *F. graminearum*. Allelic variants of *TRI* genes are responsible for the variation in structure and function of biosynthetic enzymes among different *Fusarium* species. Due to their high risk to human and animal health, the maximum tolerable limit of mycotoxins in cereals and their by-products has been regulated in many countries (Mitra Mazumder and Sasmal, 2001; Ji et al., 2014).

Proteinaceous inhibitors of microbial enzymes are central components of plant defense against a wide range of pathogens, as they protect cellular structures from degradation and/or interfere with signal transduction pathways (Ryan, 1990; Bellincampi et al., 2004; Juge, 2006). Plants may have evolved proteins that inhibit fungal phytases and thereby minimize infection and the spread of diseases. In our previous study, crude barley protein extract was able to inactivate microbial phytase activity through aspartic acid proteinase activity (Bekalu et al., 2017). Furthermore, the overexpression of nepenthesin 1 (*HvNEP-1*) in barley grains protected the barley from *Fusarium* infection and mycotoxin accumulation (Bekalu et al., 2019, 2020b). In this study, we characterized recombinant HvNEP-1 (rHvNEP-1) after expression in *Pichia pastoris*, we studied its effect on *Fusarium* growth and mycotoxin synthesis, and we uncovered that rHvNEP-1 mediates the proteolysis of *F. graminearum* and *F. culmorum* phytases.

## Materials and Methods

### Identification of Phytase Inhibitor by Mass Spectrometry (MS)

Crude protein extracts were prepared from grains of barley cv. Invictus, as previously mentioned (Bekalu et al., 2017). After being saturated to 60% with ammonium sulfate, the precipitated proteins were collected by centrifugation (7,000 × *g*, 15 min, 4°C). The protein pellet was resuspended in 50 mL of 25 mM acetate buffer (pH 4.5) and dialyzed against 50 mM Tris–HCl buffer (pH 7.5) overnight. The supernatant was collected by centrifugation (7,000 × *g*, 30 min, 4°C) and concentrated using Vivaspin Turbo 30 kDa cutoff (Sartorius, Germany). Proteins (>30 kDa) were loaded onto an ÄKTA fast protein liquid chromatography (FPLC) (GMI, MN, United States) device equipped with a Superdex G200 column, as previously mentioned (Andrews, 1964). The resulting 79 FPLC fractions were assessed for the inactivation of the *Aspergillus ficuum* phytase, as described earlier (Bekalu et al., 2017). Phytase inactivating and non-inactivating fractions were analyzed by MS (Dionisio et al., 2011).

### HvNEP-1 Amplification, Cloning, and Sequencing

The candidate aspartic protease nepenthesin 1 (*HvNEP-1*) designated under the UniProt Accession Number M0W9B2 was tblastN against the barley genomic sequence in the NCBI database^1^ and the IPK Barley BLAST server^2^. Genomic DNA (gDNA) was extracted from the leaves of 6-day-old barley cv. Invictus seedlings (Doyle and Doyle, 1990). Since the gene has no intron, the coding region (CDS) of *HvNEP-1* was PCR-amplified using gDNA as template and gene-specific primers P1 (Supplementary Table 1). PCR was carried out in a 50 μL reaction mixture containing 100 ng gDNA template (2 μL), 10 μL 5 × Herculase II Buffer, 10 μL 2 mM dNTP mixture, 2 μL primer mixture P1 (10 pmol/μL), 0.5 μL Herculase II DNA polymerase (2 U/μL), 3 μL DMSO, and 20.5 μL ddH_2_O. The PCR condition contained denaturation step at 96°C for 2 min, then 40 cycles of 96°C for 1 min, 60°C for 20 s, 72°C for 2.30 min, and final extension step at 72°C for 2.30 min. The amplified fragment of 1.5 kb was gel-purified and cloned into the pCRII-TOPO Blunt vector according to the instructions of the manufacturer (Invitrogen, Waltham, MA, United States). Selected clones were evaluated for the insert by restriction digestion and sequencing (Eurofins Genomics, Germany).

### Sequence and Phylogenetic Analysis of HvNEP-1

Multiple sequence alignment of the HvNEP-1 and related PAPs was performed with Clustal Omega. Potential N-glycosylation sites were predicted using NetNGlyc 1.0 Server^3^. Additional sequence features of HvNEP-1 such as the catalytic triads, NAP-I sequence, and flap tyrosine (Tyr) residue were annotated based on the information described for nepenthesins and homologs mentioned in the study of Athauda et al. (2004). The domains of HvNEP-1 were annotated using the Profile Hidden Markov Model (HMM) scan^4^. For phylogenetic analysis, the amino acid sequences of HvNEP-1 and related PAPs were primarily aligned with MUSCLE sequence alignment program. The tree was constructed by the neighbor-joining method using MEGA X. Nepenthesin, constitutive disease resistance 1 (CDR1), chloroplast nucleoid DNA-binding protein with 41 kDa (CND41), promotion of cell survival 1 (PCS1), and nucellin were included to represent the nepenthesin-like PAPs. Phytepsins were added as an out-group from the pepsin-like PAPs for the cluster analysis. Cluster designation of the nepenthesin-like PAPs was performed based on the information described in the earlier studies (Chen et al., 2009; Guo et al., 2013).

### Expression and Purification of rHvNEP-1 in Yeast

The KM71H strain of *P. pastoris* was used for the heterologous expression of HvNEP-1 protein. The transformation was carried out using the pGAPZαA expression vector (Invitrogen, United States). For expression, the native signal peptide (Δ*HvNEP-1*) was removed, and a hexahistidine (His_6_) residue was inserted at the C-terminal for affinity purification of the rHvNEP-1. The signal peptide cleavage site was predicted using SignalP^5^. The PCR primer P2 was designed to clone into the pGAPZαA vector by In-Fusion (Takara Bio, Japan). The PCR was performed under the abovementioned conditions, and the resulting fragment was inserted into the pGAPZαA vector digested with *Xho*I and *Sal*I restriction enzymes. The amplified PCR product was fused downstream of the alpha-mating factor secretion signal to promote the secretion of rHvNEP-1 into the cell supernatant. The *P. pastoris* glyceraldehyde-3-phosphate dehydrogenase (*GAP*) promoter was used to drive the constitutive expression of the rHvNEP-1 protein. The pGAPZαA–Δ*HvNEP-1* construct was transformed into TOP10F’ *Escherichia coli* competent cells and selected on a low-salt Luria-Bertani agar plate containing 25 μg/mL of zeocin. Plasmids were isolated from positive colonies, as recommended by the instructions of the manufacturer (MACHEREY-NAGEL, Düren, Germany). The plasmid integrity was confirmed by restriction digestion and sequencing using primers P2 and P3.

For transformation into *P. pastoris*, the intact expression plasmid was linearized with *Avr*II or *Bsp*HI restriction enzymes. Of note, 5 μg of the linearized plasmid was transformed into *P. pastoris* cells by electroporation (1.8 kV, 25 μF, 200 Ω). Transformant colonies of *P. pastoris* were selected on buffered yeast extract peptone dextrose (YPD) plates (1% yeast extract, 2% peptone, 2% dextrose (glucose), and 2% agar, pH 6.5) supplemented with 100 μg/mL of zeocin. After incubated at 28°C for 3 days, selected colonies were transferred into fresh-buffered YPD plates containing up to 400 μg/mL of zeocin. The integration of *HvNEP-1* into *P. pastoris* genome was confirmed by PCR using the primer pair P2. Selected *P. pastoris* clones were then inoculated into 200 mL of buffered YPD medium [1% (w/v) yeast extract, 2% (w/v) peptone, and 1% (v/v) glucose, pH 6.5] and cultivated at 25°C with slow shaking (140 rpm) for 4 days. The supernatant was collected by centrifugation and its pH was adjusted to 6.5 before loading. Then, it was loaded onto Ni/NTA column (Qiagen, Hilden, Germany) pre-equilibrated with the equilibration buffer (30 mM MES, pH 6.5; 20 mM NaCl). The column was washed with two column volumes of buffer (50 mM MES, pH 6.5; 300 mM NaCl; 20 mM imidazole), and the bound protein was eluted using the buffer (50 mM MES pH 6.5; 300 mM NaCl; 300 mM imidazole). The expression rHvNEP-1 in *P. pastoris* was confirmed by the matrix-assisted laser desorption/ionization time-of-flight MS (MALDI-TOF-MS), SDS–PAGE, and Western blotting. Untransformed *Pichia* was included as a negative control. The supernatant was collected and analyzed by SDS–PAGE and Western blotting.

### Protein Quantification, SDS–PAGE, and Western Blotting

Total protein concentration in *Pichia* media and purified eluates was determined by the Bradford method, using bovine serum albumin (BSA) as a standard. The expression of rHvNEP-1 was monitored by SDS–PAGE using precasted 4–12% NuPAGE gels (Life Technologies, CA, United States). Molecular weight was estimated using Precision Plus Protein^TM^ All Blue Prestained Protein Standard (BioRad, CA, United States). Western blotting was carried out using the semi-dry blotting apparatus (Hoefer, CA, United States). Anti-His mouse monoclonal antibody (Roche, Basel, Switzerland) and a goat anti-mouse immunoglobulin G (IgG) alkaline phosphatase conjugate (BioRad, CA, United States) were used for the detection of the recombinant His_6_-tagged protein. The alkaline phosphatase was detected using the conjugate substrate FAST BCIP/NBT (Sigma, MO, United States). The immature rHvNEP-1 was activated in 100 mM formate buffer (pH 2.5) incubated at 25°C for 1 h, and the activation process was terminated using 100 mM acetate buffer (pH 5.0).

### Characterization of rHvNEP-1 Activity

Proteolytic activity was measured using a modified method of Anson (Anson, 1938). Briefly, 5 μL of 1 mg/mL rHvNEP-1 was added to 100 μL of 2% hemoglobin in 100 mM acetate buffer (pH 5.0). The reaction was allowed to proceed for 1 h at 37°C and stopped by adding 100 μL of 5% trichloroacetic acid (TCA). Then, the mixture was centrifuged for 10 min at 10,000 × *g*. The absorbance of TCA non-precipitable peptides was measured at 280 nm against the sample, using a spectrophotometer (Epoch, VT, United States).

### Effect of Generic Protease Inhibitors on rHvNEP-1 Activity

The activity of rHvNEP-1 was examined in the presence of E-64 (50 μM), pepstatin A (100 μM), phenylmethylsulfonyl fluoride (PMSF, 1 mM), ethylenediaminetetraacetic acid (EDTA, 5 mM), or dimethyl sulfoxide (DMSO, 3%). The rHvNEP-1 activity assay was performed as described previously. Briefly, 5 μL of 1 mg/mL rHvNEP-1 and an inhibitor were mixed and incubated by shaking with 2% hemoglobin in 100 mM acetate buffer (pH 5.0), for 1 h at 37°C. The residual protease activity was determined as a percentage relative to their respective sample controls. Sample controls were prepared using all the components except the protease inhibitors.

The optimal conditions for rHvNEP-1 activity were presented as percent phytase activity. The phytase activity was assayed as previously described (Bekalu et al., 2017). Briefly, 5 μg of rHvNEP-1 was added to 2.5 U/mL of *A*. *ficuum* phytase (Sigma P-9792), 2 mM sodium phytate, and 400 μL of 25 mM sodium acetate buffer (pH 5.5) containing 0.1 mM CaCl_2_. After incubation at 37°C for 1 h, the reaction was ended by adding 800 μL of stop solution (i.e., 20 mM ammonium heptamolybdate, 5 mM ammonium vanadate, and 6% nitric acid). It was then centrifuged at 4,032 × *g* for 5 min, and the absorbance was measured at 415 nm using a spectrophotometer (Epoch, VT, United States).

### Optimal pH and Temperature

The optimal pH for rHvNEP-1 was determined by measuring the phytase activity after incubated at 37°C for 1 h using the buffers as follows: pH 2.0–2.5, 100 mM formate; pH 3.0–5.5, 100 mM acetate; pH 6.0–7.0, 100 mM sodium phosphate; pH 8.0, 100 mM Tris–HCl. Similarly, the optimal temperature was determined at various temperatures ranging from 20°C to 80°C in 100 mM acetate buffer (pH 5.0), after incubated for 1 h. Sample blanks were prepared without rHvNEP-1. The activity of rHvNEP-1 was calculated as percent inactivation of phytase activity in the presence of rHvNEP-1, compared with the corresponding sample blanks. The assay was performed in three technical replicates.

### pH and Temperature Stability

The pH stability of rHvNEP-1 was determined after preincubated in buffers ranging from pH 2.0 to 8.0 at 25°C for 1 h. The phytase activity was calculated as a percentage of activity in the presence of preincubated rHvNEP-1 against non-incubated rHvNEP-1, at 40°C for 1 h in 100 mM acetate buffer (pH 5.0). The thermal stability was examined after preincubation of rHvNEP-1 at 40°C to 80°C in 100 mM acetate buffer (pH 5.0) for 1–3 h. After cooled down to 25°C, rHvNEP-1 was mixed with *A*. *ficuum* phytase, and the phytase activity assay was performed at pH 5.0, 37°C for 1 h. The phytase activity was expressed as a percentage of activity in the presence of preincubated rHvNEP-1, compared with the non-incubated rHvNEP-1. The assay was performed in three technical replicates.

### Phytase Inactivation by rHvNEP-1

Phytase inactivation was investigated by incubating 100 μg of *A. ficuum* or wheat recombinant TaPAPhy_b2 (TaPAPhy) phytase with rHvNEP-1 or pepsin in the ratios of 1:0.002, 1:0.005, 1:0.01, 1:0.02, and 1:0.05 of phytase to rHvNEP-1 protease (w:w) at 25°C for 1 h. The TaPAPhy was included from the previous study (Dionisio et al., 2011) to examine the effect of rHvNEP-1 on plant phytases, and pepsin was included as a negative control. Briefly, sodium phytate substrate (2 mM) was added to the preincubated mix and incubated for an additional 1 h at 37°C. The rHvNEP-1 reactions were performed in 100 mM sodium acetate buffer (pH 5.0), whereas the pepsin reactions were performed in 50 mM formate buffer (pH 2.5). For sample blanks, the rHvNEP-1 or pepsin was replaced by an equal volume of the assay buffers. The residual phytase activity was determined as a percentage of activity in the protease-treated samples, compared with the corresponding sample blanks.

### Effects of rHvNEP-1 on *Fusarium* Phytases, Fungal Growth, and Toxin Production

Primarily, the screening of phytase-producing *Fusarium* strains and the preparation of crude phytases were performed as previously described (Gontia-Mishra et al., 2013). *F. graminearum* 7775 and *F. culmorum* 8984 strains were plated out on phytate-specific medium (PSM), containing 5 g sodium phytate, 10 g sucrose, 2 g (NH4)_2_SO_4_, 3 g tryptone, 2 g yeast extract, 0.5 g KCl, 0.5 g MgSO_4_, 0.01 g MnSO_4_⋅5H_2_O, 0.01 g FeSO_4_, 1 g Triton X-100, and 15 g/L agar, pH 7.0. Crude phytases were extracted from *F. graminearum* 7775 and *F. culmorum* 8984 strains that showed strong phytate-degrading ability on the PSM plates. Briefly, 100 mL of PSM liquid media was inoculated with spore suspension (1 × 10^5^) of *F. graminearum* 7775 or *F. culmorum* 8984. Fungal cultures were incubated at 30°C for 5 days, with shaking at 250 rpm. Supernatants were collected by centrifugation (5,000 × *g*, 10 min, 4°C) and used for phytase activity assays. Supernatant containing *Fusarium* phytases (100 μg) was incubated with rHvNEP-1 in the phytase/rHvNEP-1 protease (w:w) ratios of 1:0.002, 1:0.005, 1:0.01, 1:0.02, and 1:0.05 at 25°C for 1 h. Phytase activities for both *Fusarium* strains were determined as described for *A. ficuum* or TaPAPhy phytases. The assay was performed in three technical replicates.

The rFgPHY1 and rFcPHY1 were produced to examine the phytase-hydrolyzing properties of rHvNEP-1. The CDS of both *FgPHY1* (XP_011317825) and *FcPHY1* (PTD11210) phytases were PCR-amplified using primer P4. Sequences for the signal peptide (SP) were removed, and C-terminal 6xHis-tag was introduced for affinity purification of the recombinant phytases. The resulting sequences were cloned into the pGAPZαA expression vector digested with *Sal*I and *Xho*I by In-Fusion cloning using primer P5. Cloning, heterologous expression, and purification of the rFgPHY1 and rFcPHY1 in *P. pastoris* were performed as stated for rHvNEP-1. Positive *Pichia* clones were grown in YPD liquid media, pH 6.5; and rFgPHY1 and rFcPHY1 were purified after the pH was adjusted to 6.5. The analysis was performed by incubating 15 μg of rFgPHY1 or rFcPHY1 with 15 μg of rHvNEP-1 in 50 mM acetate buffer (pH 6.5), at 25°C, for 1 and 24 h. After incubation, the reaction was stopped by adding SDS sample buffer and examined on SDS–PAGE.

The antifungal activity of rHvNEP-1 was studied on *F. graminearum* strain JCM9873. The preparation of fungal culture and the toxin analysis were performed as described previously (Etzerodt et al., 2015). Biomass and toxin profiles were analyzed after incubation with rHvNEP-1. Briefly, 100 μL (3.47 mg) of rHvNEP-1 or 100 μL of 100 mM acetate buffer (pH 5.5) were added into 1 mL of fungal cultures (10^7^ spores) and incubated by shaking (22°C, 130 rpm) on second, third, sixth, and eighth days in the dark. On sampling days, similar amounts of rHvNEP-1 and 100 mM acetate buffer (pH 5.5) were added to the remaining cultures. For instance, on Day 2, 100 μL of rHvNEP-1 or 100 mM acetate buffer (pH 5.5) were added to third-, sixth-, and eighth-day cultures, and so on. On the respective days, mycelial mass was collected by centrifugation (15,000 × *g* at 4°C for 20 min), and toxin profiles were analyzed. The dry mass of the fungal samples was measured from freeze-dried mycelia (Banerjee et al., 1993).

### Relative Expression Analysis of *TRI* Genes

Of note, 1-mL culture of *F. graminearum* strain JCM9873 were grown with 3.47 mg of rHvNEP-1 or 100 μL of 100 mM acetate buffer (pH 5.5; control) for 2, 3, 6, and 8 days. Fungal mycelia were separated from the cultures by centrifugation (10,000 × *g*, 25°C, 10 min). Total RNA was extracted from the mycelial mass collected on Day 8 (Chomczynski and Sacchi, 2006). RNA samples were treated with DNase according to the instructions of the manufacturer (Roche, Basel, Switzerland). Reverse transcription of mRNA was performed using Superscript III-RT (Invitrogen, United States), and oligo(dT) 21T-anchor-containing primers. The expressions of *TRI4*, *TRI5*, *TRI6*, and *TRI12* were normalized using the expression of *GADPH*. The *TRI* gene-specific quantitative PCR (qPCR) primers are described in Supplementary Table 1. The qPCR was performed in a final reaction volume of 12 μL containing 6 μL Power SYBR Green Master Mix (Applied Biosystems, MA, United States), 1 μL of diluted cDNA, 2.4 μL of 1.5 μM primer mix, and 2.6 μL of sterile Milli-Q water. The samples were set up in the 384-well plates, and the qPCR was run and detected in an AB7900HT sequence detection system (Applied Biosystems, MA, United States).

### Statistical Analysis

Experiments were carried out in three biological or technical replicates. A two-way ANOVA was used to compare the data with statistical significance considered as *p* < 0.05.

## Results

### Identification of Microbial Phytase Inhibitor by MS

A total of 79 FPLC fractions were collected. Of these, six fractions with phytase inactivation and eight with no phytase inactivation were included for the MS analysis. The MS of the most inhibitory fractions identified several proteins of which HvNEP-1 appeared a likely candidate inhibitor (Supplementary Data Set), The result corresponds to the finding that phytase inactivation in the crude protein extracts of barley grains is ascribed to aspartic protease activity in the seed extract (Bekalu et al., 2017). The molecular weight of HvNEP-1 was 48.915 kDa. The summary of protein hits for the crude protein extract and the representative FPLC fractions are included in Supplementary Data set.

### Sequence Analysis of HvNEP-1

**FIGURE 1:**
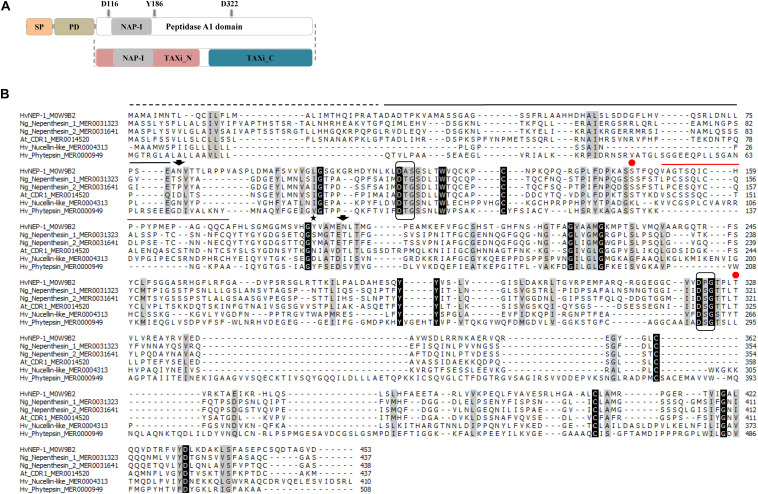
Analysis of the nepenthesin 1 (HvNEP-1) sequence. **(A)** The primary structure organization of HvNEP-1. Primary structural domains are indicated as signal peptide (SP), prodomain (PD), and the peptidase A1 domain. The nepenthesin-type plant aspartic proteases (PAP) insertion (NAP-I), catalytic Asp residues (D116 and D322), and flap Tyr residue (Y186) are located inside the peptidase A1 domain. The Profile Hidden Markov Model (HMM) scan of HvNEP-1 locates the TAXi_N and TAXi_C domains. **(B)** Multiple sequence alignment of the HvNEP-1 and related PAPs. The PAP amino acid sequences retrieved from UniProt or MEROPS database are used for the alignment as follows: (1) *Hordeum vulgare* nepenthesin 1 (M0W9B2), (2) *Nepenthes gracilis* nepenthesin 1 (MER0031323), (3) *N. gracilis* nepenthesin 2 (MER0031641), (4) *Arabidopsis thaliana* CDR1 (MER0014520), (5) *H. vulgare* nucellin (MER0004313) and (6) *H. vulgare* phytepsins (MER0000949). SP (broken line), PD (black solid line), NAP-I (red solid line), catalytic triads (boxed), flap Tyr residue (Y186) (star), putative N-glycosylation sites (arrows), and Ser35 and Thr218 (red dots). Residues are shaded black or gray depending on the level of conservation among sequences.

Using available genomic sequence in the IPK Barley BLAST server, an open reading frame (ORF) of 1,362 bp was predicted to encode a full-length HvNEP-1 protein of 453 amino acids with a predicted molecular weight of 48.9 kDa. The deduced protein encoded a preproenzyme with putative SP, prodomain (PD), and long polypeptide (peptidase A1 domain) interrupted by the NAP-I domain (Figure 1A). The primary structure exhibited the characteristic feature of nepenthesin-like PAPs, where Asp116, Tyr186, and Asp322 residues form the active site. In addition, Ser35 and Thr218 residues (i.e., in reference to pepsin numbering) were also present in the catalytic center.

Further analysis of the HvNEP-1 sequence using the Profile HMM scan identified the TAXi_N and TAXi_C xylanase inhibitor domains (Figure 1A). The predicted 3D structure of the protein displayed a catalytic pocket formed by the two catalytic Asp residue triads supported by a Tyr residue (Y186) in the flap (Supplementary Figure 1). The TAXi_N and TAXi_C domains form each lobe that contributes a catalytic Asp residue for hydrolyzing the substrate. Multiple sequence alignment of the HvNEP-1 protein sequence and related PAPs revealed that the catalytic Asp residues were conserved but not the flap Tyr residue (Figure 1B). The residues forming the catalytic triads with Asp in HvNEP-1 were also different from the characteristic PAP residues (i.e., Asp-Thr-Gly/Asp-Ser-Gly and Asp-Thr-Gly). Besides, the NAP-I sequence contained two Cys residues rather than the four common-to-most nepenthesin-like PAPs. Two potential N-glycosylation sites were predicted for HvNEP-1 (Figure 1B). Protein BLAST HvNEP-1 finds around 90% identity to aspartic proteinase nepenthesin-2-like (XP_037427783.1) and aspartic proteinase nepenthesin-1-like (XP_020183092.1) from emmer wheat and Tausch’s goat grass, respectively. The protein showed less than 20% sequence homology to nepenthesins from *Nepenthes* species.

**FIGURE 2:**
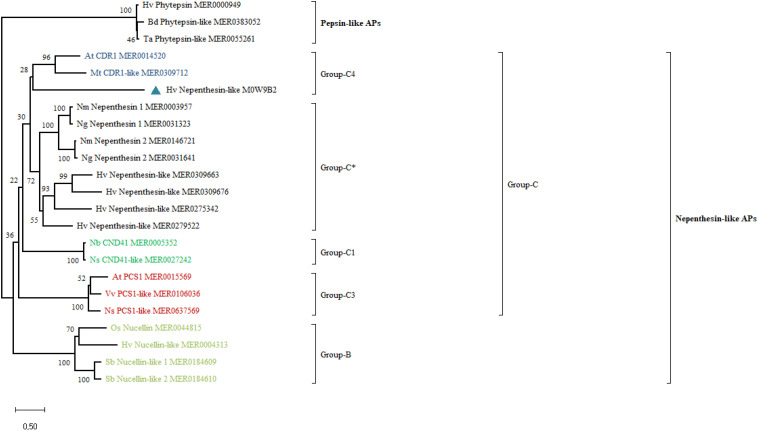
Phylogenetic analysis of HvNEP-1 and related nepenthesin-like activator proteins (APs) from various plant species. Percentage bootstrap values from 1,000 replicates are indicated at each node. The scale (0.50) represents the number of amino acid substitutions per site. Plant species included in the tree are At, *Arabidopsis thaliana*; Bd, *Brachypodium distachyon*; Hv, *Hordeum vulgare*; Mt, *Medicago truncatula*; Nb, *Nictotiana benthamiana*; Ng, *Nepenthes gracilis*; Nm, *Nepenthes mirabilis*; Ns, *Nicotiana sylvestris*; Os, *Oryza sativa*; Sb, *Sorghum bicolor*; Ta, *Triticum aestivum*; Vv, *Vitis vinifera.* MEROPS or UniProt identifiers are indicated on the tree. ^∗^ represents the most common nepenthesin cluster.

Phylogenetic analysis separately grouped the pepsin-like PAPs and subgroups of the nepenthesin-like PAPs (Figure 2). Phytepsins from the pepsin-like PAPs cluster into a separate clade. However, HvNEP-1 clustered into a separate group than other nepenthesins from barley and carnivorous species. It categorizes into the Group-C4 of the nepenthesin-like PAPs, which is represented by CDR1 (Figure 2). In this study, the clusters of representative PAPs correspond to the results published in the previous studies (Chen et al., 2009; Guo et al., 2013).

### Expression and Purification of HvNEP-1 in *P. pastoris*

**FIGURE 3:**
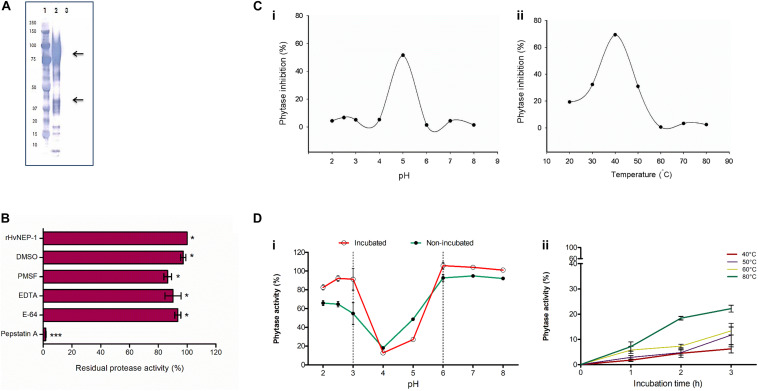
Cloning and characterization of *HvNEP-1* from grains of barley cv. Invictus. **(A)** Western blot analysis of the rHvNEP-1 protein. Lane 1: protein standard, lane 2: rHvNEP-1, and lane 3: supernatant of untransformed *Pichia* KM71H strain. Two strong protein bands that correspond to approximately 47 kDa and 92 kDa are indicated with arrows. **(B)** Residual activity of rHvNEP-1 after incubation with generic protease inhibitors. **(C)** pH **(i)** and temperature **(ii)** optima of rHvNEP-1 to inhibit the activity of *A. ficuum* phytase. Lines connect the mean of each consecutive treatments. **(D)** The effect of pH **(i)** and temperature **(ii)** on the stability of rHvNEP-1 activity. pH and temperature stabilities are described as percent residual activity of *A. ficuum* phytase in the rHvNEP-1 incubated and non-incubated samples. Values in **(B–D)** are the mean of three independent technical replicates. Error bars = mean ± SE (*n* = 3), *p < 0.05 and ***p < 0.001.

The rHvNEP-1 was successfully expressed using *P. pastoris* system. The highest rHvNEP-1 yield (0.59 mg/mL) was achieved after 3 days of growth in the YPD liquid medium, pH 6.5 on an orbital shaker (140 rpm, 25°C). Furthermore, the Western blotting confirmed the expression of rHvNEP-1, corresponding to different molecular weights (Figure 3A).

### Characterization of the rHvNEP-1 Activity

The activity of rHvNEP-1 was tested with and without specific protease inhibitors (Figure 3B). The activator protein (AP) inhibitor pepstatin A strongly inhibited the activity of rHvNEP-1 by 98.2%. PMSF, E-64, EDTA, and DMSO inhibited the activity of rHvNEP-1 by 13.5, 6.4, 9.7, and 2.7%, respectively. Incubation in a 3% DMSO caused no significant loss of enzyme activity.

The highest-level inactivation of *A. ficuum* phytase by rHvNEP-1 was noted at pH 5.0 and at 40°C (Figures 3Ci,ii). The rHvNEP-1 stability was quantified as residual phytase activity after incubation in buffers ranging from pH 2.0 to 8.0 at 25°C for 1 h (Figure 3Di). With the exception of pH 4.0 and 5.0, which showed strong activity, the rHvNEP-1 did not show improved activity for all other pH values (Figure 3Di). The increase in the activity of rHvNEP-1 at pH 5.0 was approximately two times the activity at pH 4.0 after 1 h of incubation. Under both conditions, rHvNEP-1 showed strong activity at pH values between 3 and 6, as indicated between the broken lines. The temperature stability was determined after it was incubated at various temperatures, ranging from 40 to 80°C for 1–3 h of incubation (Figure 3Dii). rHvNEP-1 showed high thermostability, as it retained its activity to inhibit 78% of phytase activity after incubation at 80°C for 3 h.

**FIGURE 4:**
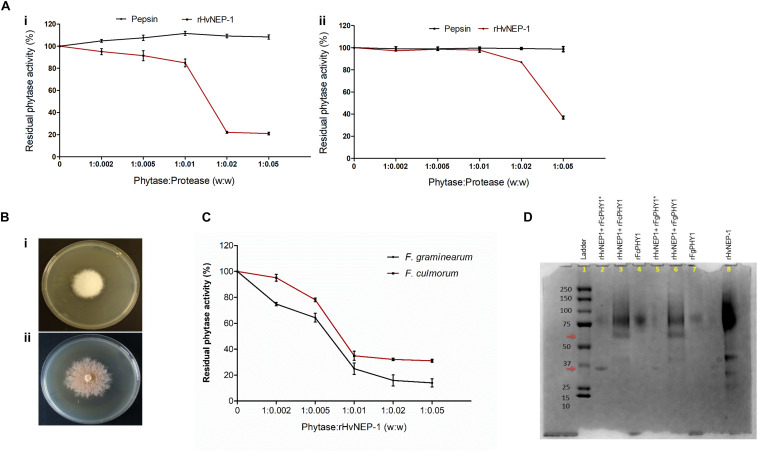
Effects of rHvNEP-1 on the activity of fungal and plant phytases. **(A)** Residual activity of the *A. ficuum* **(i)** and TaPAPhy **(ii)** phytases after incubation with rHvNEP-1 or pepsin under different phytase/protease ratio (w:w). **(B)** Screening of phytase-producing strains of *Fusarium graminearum* 7775 **(i)** and *Fusarium culmorum* 8984 **(ii)** on agar plates containing sodium phytate substrate, pH 6.5. **(C)** Activity of crude *F. graminearum* and *F. culmorum* phytases with increasing concentrations of rHvNEP-1 (w:w). Values in **(A,C)** are the mean of three independent technical replicates. Error bars = mean ± SE (*n* = 3). **(D)** SDS–PAGE analysis of rFgPHY1 and rFcPHY1 degradation by rHvNEP-1 at pH 5.0. The protein ladder indicated in lane 1. rFgPHY1 or rFcPHY1 incubated for 1 h (i.e., without asterisk, lane 3 and 6) and 24 h (i.e., with asterisk, lane 2 and 5). Lane 4, 7, and 8 stand for rFcPHY1, rFgPHY1, and rHvNEP-1, respectively. The upper and lower red arrows indicate the degradation product of rFgPHY1 and rFcPHY1 after incubated with rHvNEP-1 for 1 and 24 h.

Then, the effect of rHvNEP-1 concentrations on the activity of *A. ficuum* and wheat TaPAPhy phytases was investigated. Both phytases showed high sensitivity to rHvNEP-1, but to different extents (Figure 4A). The residual activity of the *A. ficuum* phytase started to drop at a phytase/protease ratio of 1:0.002 (Figure 4Ai). For TaPAPhy, residual activity did not drop until the phytase/protease ratio reached 1:0.005 (Figure 4Aii). In contrast, both phytases were resistant to pepsin, as the phytase activity was unaffected after it was exposed to pepsin even at a phytase/protease ratio of 1:0.05.

Both *F. graminearum* 7775 and *F. culmorum* 8984 formed a clear zone around the colonies on the PSM (Figure 4B). However, in the study of phytase activity, the addition of rHvNEP-1 reduced the activities of both crude *Fusarium* phytases from the phytase/protease ratio of 1:0.005 (Figure 4C). At all rHvNEP-1 concentrations, the activity of *F. graminearum* phytase was strongly inhibited than that of the *F. culmorum* phytase.

The effect of rHvNEP-1on the degradation of rFgPHY1 and rFcPHY1 was examined by incubating rFgPHY1 or rFcPHY1 with rHvNEP-1 for 1 and 24 h at 25°C. SDS–PAGE was employed to demonstrate the effect of rHvNEP-1 on phytases (Figure 4D). rHvNEP-1 caused significant degradation of both phytases. A clearly altered banding pattern was visible after 1 h, and most of the protein was degraded after 24 h of incubation. The result shows that both *Fusarium* phytases are liable to rHvNEP-1-mediated degradation and thereby the reduction in phytase activity.

### Antifungal Activity of rHvNEP-1

**FIGURE 5:**
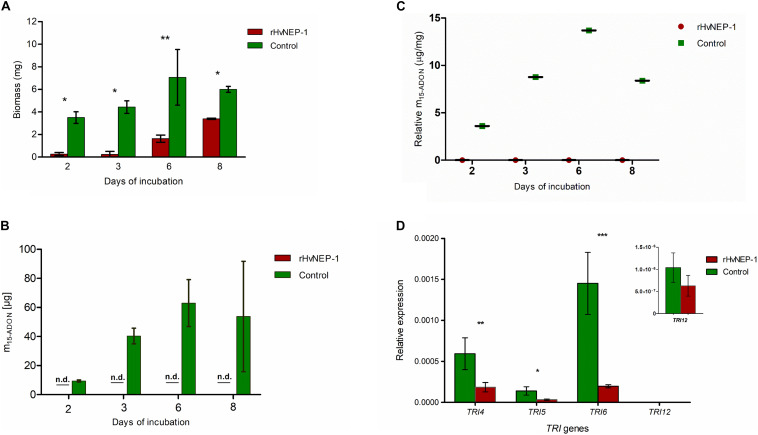
Effects of rHvNEP-1 on the biomass and mycotoxin accumulation of *Fusarium* fungi. **(A)** Comparative analysis of biomass accumulation of *F. graminearum* strain JCM9873 with and without rHvNEP-1. **(B)** *In vitro* accumulation of 15-ADON in *F. graminearum* JCM9873 strain in the presence and absence of rHvNEP-1. n.d. denotes for toxin below detection level in the rHvNEP-1-incubated cultures. Values in **(A–C)** are the mean of three independent technical replicates. **(C)** Relative accumulation of 15-ADON to fungal biomass. It is calculated from the mean of 15-ADON and fungal biomass for the sampling day. **(D)** Quantitative PCR (qPCR) analysis of *TRI* genes isolated from *F. graminearum* JCM9873 strain cultured with and without rHvNEP-1. Values are the mean of three independent biological replicates. Error bars in **(A–D)** = mean ± SE (*n* = 3). *p < 0.05, **p < 0.01, and ***p < 0.001.

Biomass growth and mycotoxin accumulation of *F. graminearum* strain JCM9873 were investigated on growth media with and without rHvNEP-1. Fungal growth was significantly suppressed by the addition of rHvNEP-1 (Figure 5A). Moreover, rHvNEP-1 significantly reduced the production of the mycotoxin 15-ADON (Figure 5B). In rHvNEP-1-treated samples, 15-ADON accumulation did not increase, despite that the fungal biomass increased (Figures 5A,B). In contrast, for the growth media lacking rHvNEP-1, both biomass and 15-ADON accumulation gradually increased (Figures 5A,B). Furthermore, the relative accumulation of 15-ADON to biomass in the presence and absence of rHvNEP-1 was evaluated (Figure 5C). Under rHvNEP-1 treatment, the relative accumulation of 15-ADON in all sampling days remained as low as zero. In the control samples, the relative toxin accumulation was registered approximately from 4 to 14 μg/mg of fungal biomass.

The relative expression of four *TRI* genes (i.e., *TRI4*, *TRI 5*, *TRI 6*, and *TRI 12*) was determined for *F. graminearum* strain JCM9873 grown with and without rHvNEP-1. rHvNEP-1 had a significant effect on the expression of *TRI4* and *TRI5* but not on *TRI12* (Figure 5D). The reduction in *TRI6* expression was highly significant, indicating that rHvNEP-1 is a strong suppressor of *TRI6*.

## Discussion

Proteinaceous inhibitors of numerous microbial enzymes have been described from different plant species (Juge, 2006; Gusakov, 2010; Yarullina et al., 2016). However, although microbial phytases are used intensively in animal nutrition and have huge industrial implications, there has been no attention given to potential plant proteinaceous inhibitors of microbial phytases. So far, the inactivation of microbial phytases by metal cations has been mainly focused (Demir et al., 2017, 2018). However, pathogens require phytase activity to utilize grain phytate-bound P, and plants require proteinaceous inhibitors of microbial phytases to protect themselves from pathogen invasion. In this study, we identified an aspartic protease HvNEP-1 as a potent inhibitor of fungal phytase activity after performing the inhibition-based purification and MS analysis of phytase inhibitory fractions. The result supports our previous study that the main inhibitor of *A. ficuum* phytase activity is an aspartic-type protease (Bekalu et al., 2017).

Similar to other PAPs, HvNEP-1 has the two catalytic Asp and other residues that are essential for its catalytic activity, such as Ser35, Tyr75, and Thr218 (i.e., in reference to pepsin numbering). Ser and Thr residues maintained the charged state of Asp residues in the catalytic center and improved the proteolytic activity (Andreeva and Rumsh, 2001). The Tyr75 residue forms the flap to enclose substrates in the active center. HvNEP-1 lacks important residues in its desired position that may compromise its activity and stability. For instance, residues forming the catalytic triads differ from the rest of PAPs, and its NAP-I sequence contains two Cys residues instead of four in most nepenthesins (Athauda et al., 2004). A change of residue(s) in the catalytic triad may result in the modification of proteolytic activity (Ekici et al., 2008). The presence of such distinct residues influences the structure, catalytic activity, and substrate specificity of HvNEP-1. Therefore, to examine their significance for proteolytic activity, site-directed mutagenesis studies should be carried out in the catalytic triads of HvNEP-1. Furthermore, the mapping of its A1 protease domain into the TAXi_N and TAXi_C xylanase inhibitory domains requires additional studies, as it may also evolve an inhibitory role against microbial xylanases (Pollet et al., 2009).

Plant proteases can play vital roles in the plant–microbe interactions (Baek and Choi, 2008). They participate in pathogen recognition, induction, and execution of defense responses or the regulation of positive and negative regulators through signaling (van der Hoorn and Jones, 2004). Phylogenetic analysis clusters the majority of nepenthesins under Group-C of PAPs. This group is represented with a large number of proteases from rice and grape (Chen et al., 2009; Guo et al., 2013). HvNEP-1 clusters under the subgroup-C4 (CDR1), which is known to participate in constitutive disease resistance through systemic acquired resistance (SAR)-mediated signaling. For instance, AtCDR1 induces local and systemic defense responses by releasing mobile peptide signals (Xia et al., 2004). AtCDR1 overexpressing *Arabidopsis* lines showed increased resistance against the virulent strains of *Pseudomonas syringae* (Xia et al., 2004). Moreover, the overexpression of OsCDR1 activates constitutive defense responses in both *A*. *thaliana* and *O. sativa* (Prasad et al., 2010). Thus, the clustering of HvNEP-1 to CDR1 requires additional *in vivo* studies to confirm its functional specialization from other nepenthesins.

Successful expression of the rHvNEP-1 was confirmed with Western blot, where protein bands with various sizes have been observed (Figure 3A). The lower arrow corresponds to the size of a monomeric HvNEP-1, whereas the above arrow could probably be the result of oligomerization and/or hyperglycosylation of HvNEP-1. The smaller bands could probably be the result of proteolysis. rHvNEP-1 needs pH 5.0 and 40°C for optimal activity against fungal phytases. This is almost similar to the pH value in the apoplast and vacuole pH (5.5) and indicates that protease plays an active role in the cell. Its high temperature stability could be credited to the presence of more Cys residues for disulfide bridge formation (Soares et al., 2019). The susceptibility of microbial phytases to proteases has been extensively studied (Rodriguez et al., 2000; Zhao et al., 2007; Promdonkoy et al., 2009). They have reported that *Aspergillus* phytases were sensitive to trypsin but not to pepsin. In this study, consistent with the previous reports, *A. ficuum* phytase is resistant to pepsin. Similarly, *F. graminearum*, *F. culmorum*, and *TaPAPhy* phytases are also resistant to pepsin. In contrast, both fungal and plant phytases are sensitive to inactivation by rHvNEP-1. However, rHvNEP-1 appears more active against the fungal phytases than the wheat phytase. As observed by SDS–PAGE, rHvNEP-1 hydrolyzed the fungal phytase during incubation. The result supports our previous study describing that the inhibition kinetics of *A. ficuum* phytase does not follow any of the four known mechanisms of enzyme inhibition (Bekalu et al., 2017). As the conventional cleavage sites for most nepenthesins, HvNEP-1 might prefer Leu, Phe, Met, Lys, Arg, and Pro residues at the P1 position in the phytase sequences (Rey et al., 2013). However, an in-depth MS analysis of HvNEP-1-cleaved peptides should be performed to identify HvNEP-1 recognition sites in the fungal phytases.

*Fusarium* species produce mycotoxins during their infection of the plant (Harris et al., 1999; Alexander et al., 2009). The disruption of *TRI* reduces the production of trichothecenes and compromises fungal virulence (Chen et al., 2019). In this study, the addition of rHvNEP-1 to the *in vitro* grown *F. graminearum* caused a significant reduction in fungal biomass and 15-ADON accumulation. The qPCR analysis of four *TRI* genes revealed that rHvNEP-1 repressed the expression of *TRI* genes involved in the biosynthesis, regulation, and transport of trichothecenes. The suppression of *TRI5* expression diminishes the conversion of isoprenoid intermediate farnesyl pyrophosphate to trichodiene that serves as the precursor compound for all trichothecenes (Hohn and Vanmiddlesworth, 1986). rHvNEP-1 also significantly affected the expression of *TRI4* that encodes for a cytochrome P450 oxygenase (Tokai et al., 2007; Alexander et al., 2009). *TRI4* is responsible for four consecutive oxygenation steps, which are indispensable to build the toxic trichothecene skeleton (Desjardins et al., 1993; Alexander et al., 2009). Due to its central role, *TRI4* has been the target to develop highly specific inhibitors of trichothecene biosynthesis. Furthermore, though not significantly, rHvNEP-1 also affects the expression of the major trichothecene transporter, *TRI12* (Alexander et al., 1999). Finally, yet importantly, rHvNEP-1 significantly reduces the expression of *TRI6* that regulates the transcription of *TRI* genes involved in the synthesis and transport of DON. The global transcriptional regulator, *TRI6*, binds to the promoters of structural and regulatory genes associated with nitrogen, carbon, and lipid metabolism and also modulates their transcription (Nasmith et al., 2011). The significant effect of rHvNEP-1 on *TRI6* has substantially reduced the accumulation of fungal biomass and the production of 15-ADON in *F*. *graminearum*. Reduced metabolism of essential compounds as the result of repressed expression of *TRI6* follows the significant reduction in fungal biomass induced by rHvNEP-1. Overall, rHvNEP-1 significantly reduces the conversion of farnesyl pyrophosphate to trichodiene and trichodiene to trichothecenes (Tokai et al., 2007), and, it also minimizes the efflux of synthesized trichothecenes (Alexander et al., 1999).

## Conclusion

This study reports for the first time how a plant protease (HvNEP-1) inhibits fungal phytase activity by proteolysis. The degradation of fungal feed phytases by plant NEPs may potentially have a major negative effect on phosphate digestibility in feed. The consequence of such a potential inactivation needs to be studied further, *in vivo*. Genetic resistance is an effective strategy for managing most diseases. However, at present, few sources of genetic resistance have been reported for FHB. Antifungal genes would be of considerable interest for the development of FHB-resistant crops, and based on this study, NEP-1 protease constitutes a strong candidate as such a resistance gene.

## Data Availability Statement

The original contributions presented in the study are included in the article/Supplementary Material, further inquiries can be directed to the corresponding author/s.

## Author Contributions

ZB performed most of the experiments, data analysis, and manuscript preparation. CM participated in the *HvNEP-1* cloning and critical review of the manuscript. GD contributed to the MS identification of *HvNEP-1* and heterologous expression of rHvNEP-1 in *P. pastoris*. TE and IF contributed to the *in vitro* mycotoxin analysis of fungal samples. HB-P developed the study concept, designed the experiment, and performed manuscript writing and editing. All authors contributed to the article and approved the submitted version.

## Conflict of Interest

The authors are co-inventors of patent WO 2019/057845 A1.

## Publisher’s Note

All claims expressed in this article are solely those of the authors and do not necessarily represent those of their affiliated organizations, or those of the publisher, the editors and the reviewers. Any product that may be evaluated in this article, or claim that may be made by its manufacturer, is not guaranteed or endorsed by the publisher.

## Footnotes

1
https://www.ncbi.nlm.nih.gov/

2
https://webblast.ipk-gatersleben.de/barley_ibsc/

3
http://www.cbs.dtu.dk/services/NetNGlyc/

4
https://www.ebi.ac.uk/Tools/hmmer/search/hmmscan

5
http://www.cbs.dtu.dk/services/SignalP/
