# Supplementary material for: Barley Nepenthesin-Like Aspartic Protease HvNEP-1 Degrades Fusarium Phytase, Impairs Toxin Production, and Suppresses the Fungal Growth
Source: Front Plant Sci. 2021 Jul 29;12:702557. doi: 10.3389/fpls.2021.702557 (PMC8358834; doi:10.3389/fpls.2021.702557)
Supplement: Supplementary file 2 [file Data_Sheet_2.docx]

**Barley nepenthesin-like aspartic protease HvNEP-1 degrades *Fusarium* phytase, impairs toxin production and suppresses the fungal growth**

Zelalem Eshetu Bekalu^1^, Giuseppe Dionisio^1^, Claus Krogh Madsen^1^, Thomas Etzerodt^1^, Inge S. Fomsgaard^1^ and Henrik Brinch-Pedersen^1^

^1^ Department of Agroecology, Research Center Flakkebjerg, Aarhus University, DK-4200 Slagelse, Denmark

**Supplementary information**


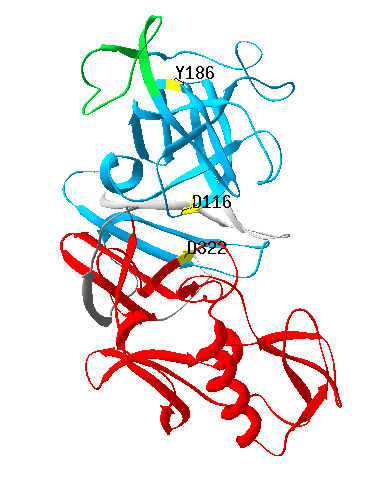

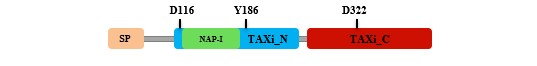


**Figure 1:** The predicted tertiary structure of HvNEP-1. Structure prediction and annotation was performed using the Swiss-Model (<https://www.swissmodel.expasy.org/>) and Swiss PDB viewer (<https://spdbv.vital-it.ch/>), respectively. The Taxi_N and Taxi_C domains from the profile-HMM scan of the HvNEP-1 sequence. Both domains are shown corresponding to the primary structure (under). Likewise, active site residues (D116, Y186 and D322), the flap Tyr residue (Y186) and the NAP-I are marked with the same color or text.

| **Table 1** Primers used in PCR, Cloning and qPCR | | | | |
| --- | --- | --- | --- | --- |
| **Gene name** | **Usage** | **Primer name** | **Primer sequence (5’-3’)** | **References** |
| HvNEP-1 | PCR | P1 | Fw: GCACATCAATGGCTATGGCGATCATGAACAC/ Rv: GTTCAATCCACACCGGCGGTGTCCTGAG | This study |
| HvNEP-1 | Cloning | P2 | Fw: GAAGGGGTATCTCTCGAGAAAAGAGCTGATGCGGACACCCCAAAAGTC  Rv: TGATGATGATGGTCGACTTAATGATGATGATGATGATGTGGATCCACACCGGCGGTGTCCTGAG | This study |
| AOX1 | Sequencing | P3 | Fw: GACTGGTTCCAATTGACAAGC / Rv: GCAAATGGCATTCTGACATCC | ^1^ |
| FgPHY1/  FcPHY1 | Sequencing | P4 | Fw: ATGATAATCAGCACCATCGCG/ Rv: TCATACAAAACATCGATCCCAGTGTCC | This study |
| FgPHY1/  FcPHY1 | Cloning | P5 | Fw: GAAGAAGGGGTATCTCTCGAGAAAAGAGAGGCTGAAGCTGAAGTCTGGGTTCACAATTACACTTTGACTTC  Rv: ATGAGTTTTTGTTCTAGATTAATGATGATGATGATGATGTGGTACAAAACATCGATCCCAGTGTCC | This study |
| TRI4 | qPCR | Tri4 | Fw: TATTGTTGGCTACCCCAAGG/ Rv: TGTCAGATGCGCCTTACAAA | ^2^ |
| TRI5 | qPCR | Tri5 | Fw: TGAAAAGGTCAAGGATCAGGA/ Rv: CCTGCTCAAAGAACTTGCAGA | ^2^ |
| TRI6 | qPCR | Tri6 | Fw: TTACATGGAGGCCGAATCTC/ Rv: AGACGCAACTCGATCAAAGAG | ^2^ |
| TRI12 | qPCR | Tri12 | Fw: ATTCGGTACATGGGCT/ Rv: GCAGTGCTGTTCGTCA | ^2^ |
| GADPH | qPCR | Gpd | Fw: CTCCCTCAACGACAACTTCG / Rv: GGAGTAACCCCACTCGTTGTC | ^2^ |

**References**

(1) Xuan, Y. J.; Zhou, X. S.; Zhang, W. W.; Zhang, X.; Song, Z. W.; Zhang, Y. X. An Upstream Activation Sequence Controls the Expression of AOX1 Gene in Pichia Pastoris. *FEMS Yeast Res.* **2009**, *9* (8), 1271–1282. https://doi.org/10.1111/j.1567-1364.2009.00571.x.

(2) Boutigny, A. L.; Barreau, C.; Atanasova-Penichon, V.; Verdal-Bonnin, M. N.; Pinson-Gadais, L.; Richard-Forget, F. Ferulic Acid, an Efficient Inhibitor of Type B Trichothecene Biosynthesis and Tri Gene Expression in Fusarium Liquid Cultures, **2009**. https://doi.org/10.1016/j.mycres.2009.02.010.
